# Neuronal Ceroid Lipofuscinosis in Border Collie Dogs in Japan: Clinical and Molecular Epidemiological Study (2000–2011)

**DOI:** 10.1100/2012/383174

**Published:** 2012-07-31

**Authors:** Keijiro Mizukami, Takuji Kawamichi, Hiroshi Koie, Shinji Tamura, Satoru Matsunaga, Shigeki Imamoto, Miyoko Saito, Daisuke Hasegawa, Naoaki Matsuki, Satoshi Tamahara, Shigenobu Sato, Akira Yabuki, Hye-Sook Chang, Osamu Yamato

**Affiliations:** ^1^Laboratory of Clinical Pathology, Department of Veterinary Medicine, Kagoshima University, 1-21-24 Korimoto, Kagoshima, Kagoshima 890-0065, Japan; ^2^Japan Border Collie Health Network, 1-14-8 Manabigaoka, Tarumi-ku, Hyogo, Kobe 655-0004, Japan; ^3^Department of Veterinary Medicine, College of Bioresource Sciences, Nihon University, 1866 Kameino, Kanagawa, Fujisawa 252-0880, Japan; ^4^Tamura Animal Clinic, 7-16 Yoshimien, Saeki-ku, Hiroshima, Hiroshima 731-5132, Japan; ^5^Japan Animal Referral Medical Center, 2-5-8 Kuji, Takatsu-ku, Kanagawa, Kawasaki 213-0032, Japan; ^6^Shinjo Animal Hospital, 104-1 Katsuragi, Nara, Katsuragi 639-2144, Japan; ^7^Laboratory of Veterinary Surgery II, School of Veterinary Medicine, Azabu University, 1-17-71 Fuchinobe, Kanagawa, Sagamihara 229-8501, Japan; ^8^Department of Veterinary Science, Nippon Veterinary and Life Science University, 1-7-1 Kyounan-chou, Tokyo, Musashino 180-8602, Japan; ^9^Department of Veterinary Clinical Pathobiology, Graduate School of Agricultural and Life Sciences, The University of Tokyo, 1-1-1 Yayoi, Tokyo, Bunkyo-ku 113-8657, Japan; ^10^Sato Animal Hospital, 6-38-20 Goinishi, Chiba, Ichihara 290-0038, Japan

## Abstract

Neuronal ceroid lipofuscinosis (NCL) is an inherited, neurodegenerative lysosomal disease that causes premature death. The present study describes the clinical and molecular epidemiologic findings of NCL in Border Collies in Japan for 12 years, between 2000 and 2011. The number of affected dogs was surveyed, and their clinical characteristics were analyzed. In 4 kennels with affected dogs, the dogs were genotyped. The genetic relationships of all affected dogs and carriers identified were analyzed. The survey revealed 27 affected dogs, but there was a decreasing trend at the end of the study period. The clinical characteristics of these affected dogs were updated in detail. The genotyping survey demonstrated a high mutant allele frequency in examined kennels (34.8%). The pedigree analysis demonstrated that all affected dogs and carriers in Japan are related to some presumptive carriers imported from Oceania and having a common ancestor. The current high prevalence in Japan might be due to an overuse of these carriers by breeders without any knowledge of the disease. For NCL control and prevention, it is necessary to examine all breeding dogs, especially in kennels with a high prevalence. Such endeavors will reduce NCL prevalence and may already be contributing to the recent decreasing trend in Japan.

## 1. Introduction

Neuronal ceroid lipofuscinosis (NCL) is a rare group of inherited, neurodegenerative lysosomal storage diseases characterized histopathologically by the abnormal accumulation of ceroid- or lipofuscin-like autofluorescent lipopigments in neurons, retinal cells, and other visceral cells throughout the body [1–4]. NCL shares certain clinical features in both human beings and animals, including behavioral abnormalities, such as personality changes and aggressiveness, mental retardation and/or dementia; motor disturbances, such as ataxia and incoordination; visual problems leading to central and/or retinal blindness; premature death, but these differ in degree based on the causative gene, of which there are currently at least 8, all recessively inherited [4]. NCL has been described in several domestic species and occurs most commonly in dogs [1, 2, 5].

NCL in Border Collies was first identified in Australia in the 1980s [6–8], and a sporadic case with the disease was also reported in the USA in the 1990s [9]. A diagnosis of the first case in Japan was made in a Border Collie that was born in 2000 [10]. The pathogenic mutation was reported in 2005 to be a nonsense mutation (c.619C>T) in exon 4 in the canine *CLN5* gene [11], which enabled a DNA-based genotyping of affected dogs and carriers. Recently, several types of rapid genotyping assays for this mutation were developed, and the carrier frequency (8.1%) in Japan was determined by a genotyping survey using these assays, suggesting the mutant allele frequency of NCL in Border Collies is high enough in Japan that measures to control and prevent the disease would be warranted [5].

The present study describes the clinical and molecular epidemiologic findings of NCL in Border Collies in Japan for 12 years, between 2000 and 2011. This study also discusses the control and prevention of the disease based on the results of these analyses.

## 2. Materials and Methods

### 2.1. Dogs Affected with NCL

The number of affected dogs was surveyed based on the records in our laboratory, which have been exclusively supporting the diagnosis of the disease in Japan. Among 27 affected dogs identified in the present study, NCL was diagnosed definitively in 25 dogs by a genetic test [5] using their specimens containing DNA, but in the remaining 2 dogs that died without any specimens before the genetic test got available, NCL was strongly suspected based on their typical clinical history and blood relationship with molecularly defined affected littermates and/or carrier parents. The clinical characteristics were analyzed and summarized using information about all of the affected dogs, which was gleaned from interviews and questionnaires of their owners and veterinarians. Some of the affected dogs were examined using the following prediagnostic tests: magnetic resonance image (MRI) scan in 7 dogs, including a previously reported dog [10]; computed tomography (CT) scan in 2 dogs; ophthalmologic examination in 3 dogs. The findings of these examinations were also analyzed and summarized.

### 2.2. Genotyping Survey

Border Collies belonging to 4 special kennels (A, B, C, and D), which generated one or more affected dogs, were surveyed between 2008 and 2010 using a genetic test with the breeders' cooperation. The number of dogs examined was 82, including 23, 20, 29, and 10 in kennels A, B, C, and D, respectively. Whole blood or saliva specimens were collected from these dogs using the Flinders Technology Associates filter paper (FTA card, Whatman International). Genotyping was performed as reported previously [5].

### 2.3. Pedigree Analysis

Pedigree analysis was performed to elucidate the genetic relationships of affected and carrier dogs identified in Japan and to deduce the pathway of transmission and distribution of the mutant allele. The genetic relationships among affected and carrier dogs found in the present study were analyzed using the pedigree papers issued by the Japan Kennel Club (http://www.jkc.or.jp/) and the Kennel Club of Japan (http://www.kcj.gr.jp/). The pedigree information of carrier dogs identified in the previous random survey in Japan [5] was analyzed and added to the results of the present study. The pedigree information of the dogs was used under the informed consent of their owners. The ancestry in Oceania was traced using pedigree information of carrier dogs provided by the New South Wales Border Collie Club (http://www.mybcsite.com/bccnswwebfiles/bccnswframe. htm), which was also disclosed via the website of the Japan Border Collie Health Network (http://www.jbchn.net/cl_carrier.htm), a volunteer breeders' association for the healthy breeding of Border Collies. Pedigree information published in the Border Collie Database (http://db.kennel.dk/) was used to supplement the information about the ancestor dogs in Oceania.

All animals were cared for and were used in the experiments in accordance with the guidelines for proper conduct of animal experiments issued by the Science Council of Japan (http://www.scj.go.jp/en/animal/index.html). All experimental procedures using animals and their specimens were performed in accordance with the guidelines regulating animal use at Kagoshima University.

## 3. Results

### 3.1. The Number and Clinical Characteristics of Border Collies with NCL

The survey revealed that there were 27 affected dogs (13 males and 14 females) in the 12 years from 2000 to 2011 in Japan (Table 1). The first case, born in 2000, was diagnosed with NCL histopathologically [10], which was confirmed molecularly using the stored liver specimen. Since this case, several affected dogs from a single or a few litters were diagnosed nearly every year up until recently in Japan. However, currently there seems to be a decreasing trend based on the observation that only 1 affected dog was identified since 2009.

The clinical features of the affected dogs are summarized and listed in Table 2. They were divided into 3 stages, that is, early (15–20 months of age), middle (19–23 months of age), and late-to-terminal stages (22–32 months of age). The overlaps in age between adjacent stages were due to individual variability in the onset and progression rate of the disease. The features at the early stage were mainly behavioral problems that most owners of the dogs did not regard as pathological changes. The features at the middle stage included serious behavioral abnormalities, visual impairments, and slight motor disorders, which prompted the owners to consult veterinarians. The features at the late-to-terminal stage included serious motor, visual, psychointellectual, and vital dysfunctions due to a wide spectrum of brain dysfunctions. The mean life span of the 23 affected dogs examined was 26.4 months of age. Of these, 17 died naturally at a mean age of 26.7 months, ranging from 23 to 32 months. The other 6 dogs were euthanized at the terminal stage an average of 25.6 months. Although there were few specific changes before 15 months of age, some of the owners had an impression that their dogs had unusual characteristics and behaviors and low learning ability in the puppy and juvenile years. There was no abnormal change on general clinicopathological examinations. No abnormal vacuoles in the cytoplasm of leukocytes were observed in blood smear examinations.

### 3.2. MRI, CT, and Ophthalmologic Examinations

**Figure 1:**
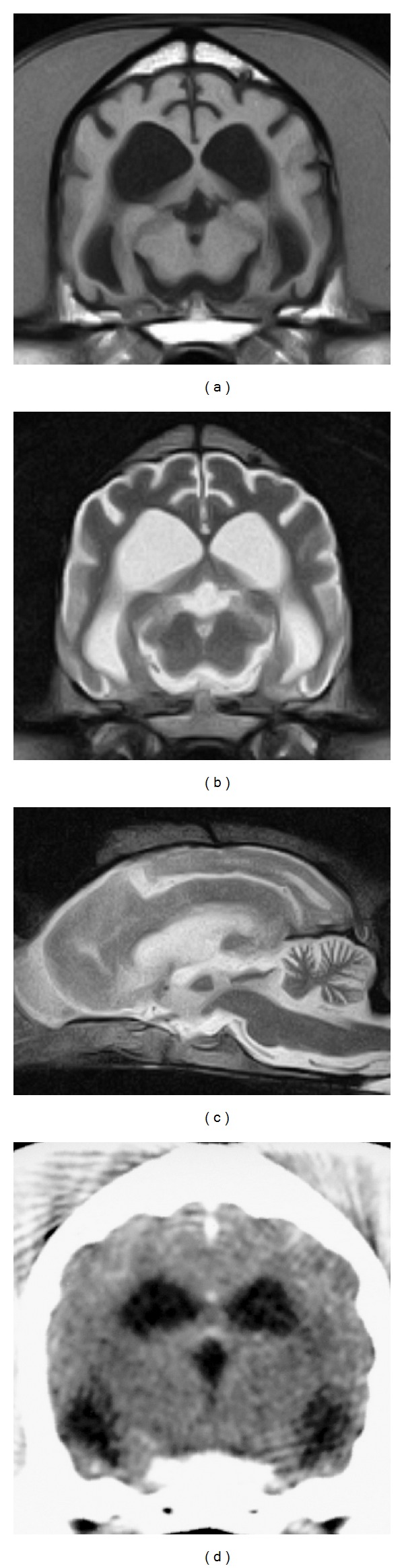
Representative magnetic resonance (MR) and computed tomography (CT) images of the brain of Border Collies with neuronal ceroid lipofuscinosis. MR images were obtained using a 0.3-tesla system (AIRIS2-comfort, Hitachi Medical Corporation) in a 24-month-old dog under general anesthesia. CT images were obtained using a multislice CT system (ECLOS, Hitachi Medical Corporation) in another 24-month-old dog under general anesthesia. (a) MR T1-weighted image (TR/TE = 500/20 ms) of a transverse section at the level of the thalamus, (b) MR T2-weighted image (TR/TE = 4,000/120 ms) of a transverse section at the level of the thalamus, (c) MR T2-weighted image of a sagittal section, and (d) CT image of a transverse section at the level of the thalamus. These images show enlarged ventricles and dilated cerebral and cerebellar sulci, suggesting forebrain atrophy.

**Figure 2:**
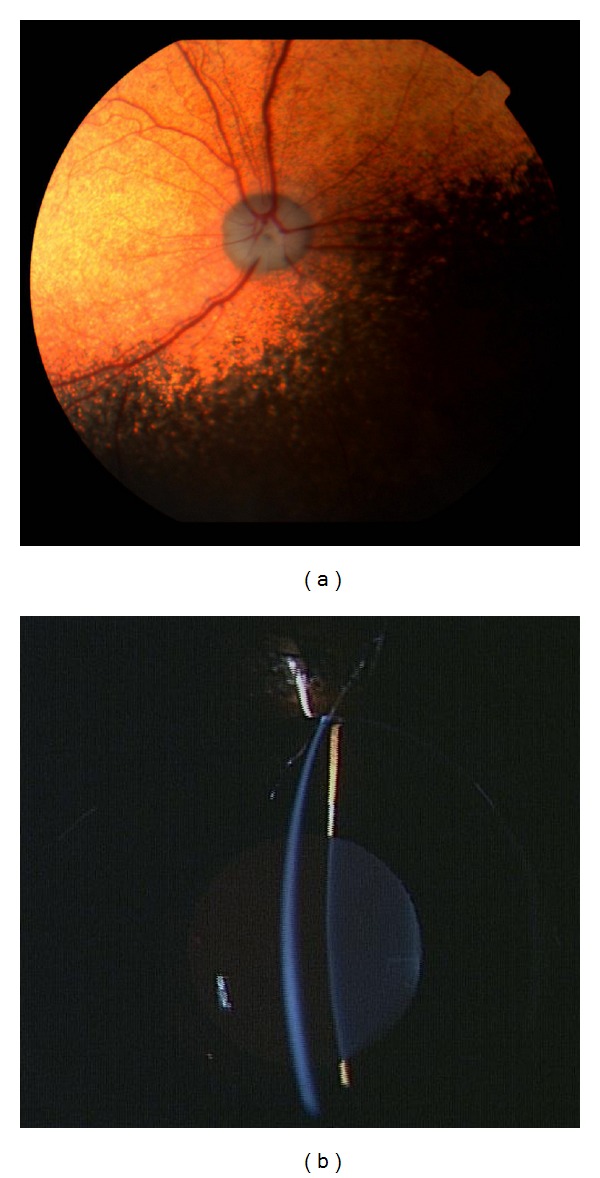
Photographs of ophthalmoscopic and slit-lamp examinations of the right eye in a 21-month-old Border Collie with neuronal ceroid lipofuscinosis. (a) Slight narrowing of blood vessels in the retina is observed, but (b) there is no abnormal finding in the slit-lamp examination.

MRI, CT, and ophthalmologic examinations were performed in affected dogs in the middle-to-late stage (at approximately 2 years of age). The common findings of MR and CT images were ventricular enlargement and well-demarcated cerebral sulci (Figure 1). Dilation of cerebellar fissures was also observed at a sagittal section of the MRI scan (Figure 1(c)). No other organic lesion was detected in MRI and CT scans. In the affected dogs examined ophthalmologically, slight narrowing of blood vessels in the retina was commonly observed in ophthalmoscopy (Figure 2(a)), but this ophthalmoscopic finding was evaluated within the normal range by veterinary ophthalmologists. An examination using a slit lamp detected no abnormal finding, such as clouding of the cornea and lens (Figure 2(b)).

### 3.3. Genotyping Survey

The results of the genotyping survey in the 4 specific kennels are shown in Table 3. The frequencies of carriers (32.9%), affected dogs (18.3%), and the mutant allele (34.8%) were markedly high in the 4 kennels.

### 3.4. Pedigree Analysis

**Figure 3:**
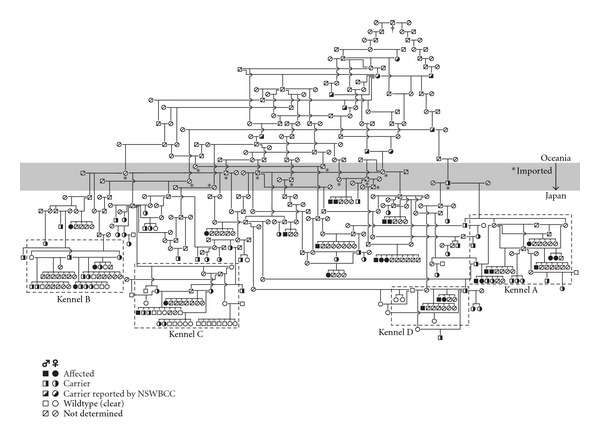
Genetic relationship of affected and carrier dogs identified in Japan between 2000 and 2011. The analysis was carried out using pedigree papers issued by the Japan Kennel Club and the Kennel Club of Japan and information reported by the New South Wales Border Collie Club (NSWBCC) and the Border Collie Database. The gray area indicates a border between Oceania and Japan. Dogs imported from Oceania to Japan were marked with an asterisk (*). All dogs carrying the mutant allele shared a common ancestor (^†^) born in 1944 in Australia. Areas surrounded by a dashed line indicate the 4 kennels surveyed using a genotyping test.

The pedigree analysis was performed mainly using the pedigree information of the 27 affected dogs and 58 carriers identified so far in Japan (Figure 3). These dogs were related to at least 13 possible carriers imported from Oceania to Japan in the middle 1990s, which ultimately revealed an ancestry from a male dog born in 1944 in Australia through 9 carriers reported by the New South Wales Border Collie Club. As a result, this analysis revealed that all dogs carrying the mutant allele in Japan share a blood relationship and a single common ancestor in Australia.

The pedigree analysis also revealed that breeding was repeated using a small number of carriers and their offspring in the 4 kennels surveyed in the present study. The breeders of these kennels were not aware of NCL in Border Collies before the genotyping survey. As a result, these kennels had markedly high frequencies of the mutant allele.

## 4. Discussion

The present study revealed 27 Border Collies (13 males and 14 females) with NCL in Japan during 2000–2011 (Table 1), although there might have been additional affected dogs elsewhere that had no opportunity to be diagnosed. Since the first affected dog, born in 2000, was diagnosed in 2002 [10], several affected dogs from a single or a few litters have been diagnosed nearly every year up until recently, but currently there seems to be a decline in the frequency of NCL. The decreasing trend may be due to the publication of the pathogenic mutation in 2005 [11], the subsequent development of a variety of genetic testing methods [5], and genotyping surveys in a random population of breeding Border Collies [5] and in a few specific kennels that generated affected dog(s) (present study). These events may have contributed to the prevention and control of the disease through the education of related breeders and fanciers of Border Collies.

Based on information from the owners and veterinarians of the affected dogs, their characteristic clinical features were determined (Table 2), although there were individual differences in the onset and progression rate of the disease and the symptoms, as reported previously [6]. Mild clinical signs, such as behavioral problems, begin at 15 months of age at the earliest, but the onset is usually a few months later (approximately 18 months of age) at the early stage. The earliest age of onset in the present study is consistent with that in a previous report [8]. Clinical signs observed at the early stage (15–20 months of age) are mainly mild behavioral abnormalities that the owners, without any knowledge of NCL, hardly regard as pathological. Visual disorders become unambiguous and behavioral abnormalities increase in severity at the middle stage (19–23 months of age). Serious clinical signs, such as convulsive seizure and motor disorders, appear at the late-to-terminal stage (older than 22 months of age). The mean life span of affected dogs was 26.7 months of age in the present study. The life span was 26.4 months of age in the dogs that died naturally, ranging from 23 to 32 months of age. This is longer than the 23.1-month life span of affected Border Collies in a previous report in Australia [7]. The difference could be attributed to the fact that many Japanese pet owners feel that euthanasia is inhumane and do not want to kill animals before they die naturally.

The characteristic clinical features determined in the present study are similar to those in previous reports [6, 7]. However, a previously reported clinical sign, mania [7], was not observed in the present study. In addition, some affected dogs had unusual characteristics and behaviors and low learning ability in the puppy and juvenile years, which was not reported previously. These clinical characteristics, updated in detail, will be of further help for the diagnosis of NCL in Border Collies.

In the MRI examination, ventricular enlargement and dilated cerebral and cerebellar sulci were common findings in affected dogs (Figure 1). The CT examination also demonstrated ventricular enlargement. These characteristic findings suggest atrophy of the forebrain, which was similarly observed in previous MRI [10] and CT examinations [9]. The changes suggesting brain atrophy developed as early as the middle stage, when affected dogs were usually referred to animal hospitals. Similar changes have been observed in other types of NCL [12, 13] and other lysosomal storage diseases, such as GM1 gangliosidosis [14] and GM2 gangliosidosis [15–17] in dogs and cats. The brain atrophy usually develops as a specific and severe change even at the middle stage in NCL but as a secondary and mild change in the late-to-terminal stage in other lysosomal diseases. Therefore, the atrophic changes would be useful as an adjunct to the diagnosis of NCL. No other organic lesion was detected in MRI scans in Border Collies with NCL, although hyperintensity in T2-weighted images of the cerebral white matter is observed in GM1 and GM2 gangliosidoses in dogs and cats [14, 16–19]. Meningeal thickening, reported in Chihuahuas with NCL [13], was not observed in Border Collies with NCL.

Visual impairments appeared to various degrees in all affected dogs, especially at the middle stage (Table 2). Previous reports have not described any abnormal changes on ophthalmoscopic examination [7, 10]. In the present study, the affected dogs examined had slight narrowing of blood vessels in the retina (Figure 2(a)), but this ophthalmoscopic finding was considered within the normal range by veterinary ophthalmologists (Figure 2). In humans with NCL, there is a pronounced loss of photoreceptors in the end stage of the disease, but in NCL in English Setters only minimal structural damage is observed in the retina [20]. In Border Collies with NCL, inclusions with variable ultrastructure are common in all cells of the retina, but the pigment accumulation does not damage the retinal architecture [8]. The retinal lesions in the Border Collies are similar to those in the English Setters but are much less severe than in juvenile NCL in humans. The narrowing of retinal blood vessels observed in affected Border Collies may be an indication of mild retinal degeneration, but this feature cannot be used as a valuable diagnostic for NCL.

Based on data of the pedigree analysis, affected dogs and carriers identified in Japan share a blood relationship and a single common ancestor born in 1944 in Australia via some presumptive carriers in Oceania (Figure 3). Although it is impossible to demonstrate whether this ancestor was a founder of NCL in Border Collies, there is a possibility that the mutant allele was transmitted from Oceania to Japan in this way mainly in the middle of the 1990s, when the causative mutation had not been identified yet. The recent increase in prevalence of the disease in Japan is likely due to an overuse of imported carriers and their offspring as breeding dogs without any knowledge of the disease. Hereafter, dogs that will be bred should be genotyped beforehand.

The pedigree analysis also suggests that breeding has been repeated using a small number of carriers and their offspring in the 4 kennels that generated affected dogs (Figure 3). There was a trend toward inbreeding in these kennels. In addition, the breeders of the kennels were not aware of the disease until they were informed of the generation of affected dogs in their kennels. These issues may have caused the high frequencies of carriers (32.9%) and the mutant allele (34.8%) in the 4 kennels (Table 3), compared to the carrier (8.1%) and mutant allele frequencies (4.1%) in the random population of Border Collies [5]. Fifteen (55.6%) of the 27 affected dogs identified so far in Japan were generated in these 4 kennels. It is thought that affected dogs are generated in a small proportion of kennels, which have a high frequency of the mutant allele. Therefore, it is important for prevention of the disease to rapidly genotype all breeding dogs in kennels that have had an opportunity to generate affected dogs. This type of examination helps the kennels not only stop generating additional affected dogs but also stop spreading carriers to other kennels. In addition, a genotyping test using specimens from a random population of breeding Border Collies should be continued [5] to detect sporadic carriers and prevent them from being used as breeding dogs. These approaches would gradually decrease the number of dogs carrying the mutant allele. These active and continuous preventive measures may be necessary to eliminate NCL in Border Collies.

## Figures and Tables

**Table 1:** Number of Border Collies with neuronal ceroid lipofuscinosis and the number of litters that included affected dogs during the 12 years between 2000 and 2011 in Japan.

Year of birth	Number of affected dogs (male and female)	Number of litters
2000	2 (2, 0)	1
2001	6 (3, 3)	3
2002	0 (0, 0)	0
2003	4 (3, 1)	3
2004	4 (1, 3)	2
2005	2 (1, 1)	2
2006	1 (0, 1)	1
2007	2 (1, 1)	2
2008	5 (2, 3)	4
2009	1 (0, 1)	1
2010–present^∗^	0 (0, 0)	0

Total	27 (13, 14)	19

*June 2012.

**Table 2:** Summary of clinical features in Border Collies with neuronal ceroid lipofuscinosis^∗^.

Stage (months of age)	Clinical signs
Early (15–20)	Altered characteristics; disregard for owner's commands; loss of interest in play and other dogs; morbid fear of noise, humans, and unspecified things; hallucination; disorientation; biting; averse to going up and down stairs (especially down)

Middle (19–23)	Fly-biting behavior; continuous shaking of the head; sudden halts during walks; uncooperativeness with other dogs; head tilt; chomping without food; tooth grinding; leg jerking; visual impairments (fear of darkness, unawareness of things such as food, and frequently hitting obstacles); aggressiveness; excitation; staggering; falling; toileting accidents; myoclonus; myoclonic seizure

Late to terminal (22–32)	Wandering; hair-pulling disorder; circadian rhythm disorder; acoustic and cutaneous hyperesthesia; cognitive and emotional impairments; blindness; dysmetria; gait deficiency; convulsive seizure; face and mouth tic; chewing difficulty; lethargy; stupor; death (mean age 26.7 months, ranging from 23 to 32 months)^†^

*Data  summarized from the information of 27 affected dogs.

^†^Data from 17 affected dogs that died naturally without euthanasia.

**Table 3:** Results of the genotyping survey carried out in 4 kennels that generated affected dogs^∗^.

Kennel	Number of			Frequency (%)		
	Dogs examined	Carriers	Affected dogs	Carriers	Affected dogs	Mutant allele
A	23	9	8	39.1	34.8	54.3
B	20	11	2	55.0	10.0	37.5
C	29	6	2	20.7	6.7	17.2
D	10	1	3	10.0	30.0	35.0

Total	82	27	15	32.9	18.3	34.8

*These genotyping surveys were carried out between 2008 and 2010.
